# Characteristics, management and outcomes of patients with hyponatraemia presenting to an Irish tertiary hospital

**DOI:** 10.1530/EC-24-0666

**Published:** 2025-03-20

**Authors:** Aoife Courtney, Lok Yi Joyce Tan, Ali Elsheikh, Faye O’Donovan, Iman Faez, Rachel Riegel, Tara O’Sullivan, Antoinette Tuthill, Oratile Kgosidialwa

**Affiliations:** ^1^ Department of Endocrinology and Diabetes Mellitus, Cork University Hospital, Cork, Ireland; ^2^ School of Medicine, University College Cork, Cork, Ireland

**Keywords:** hyponatraemia, sodium, syndrome of inappropriate ADH

## Abstract

**Objective:**

Hyponatraemia is the most common electrolyte abnormality in clinical practice and is associated with increased in-hospital mortality and length of stay (LOS). The aims of this study were to evaluate the prevalence of hyponatraemia in adult medical inpatients, compliance with consensus guidelines regarding evaluation and management of hyponatraemia, LOS and mortality.

**Design:**

This was a retrospective single-centre observational study conducted in a tertiary-level Irish hospital.

**Methods:**

Adult patients admitted under the medical services over a 2-month period with a serum sodium (sNa) <135 mmol/L were included. Patients were classified according to nadir sNa during their admission; mild, moderate and severe hyponatraemia were defined as an sNa between 130 and 134 mmol/L, 125–129 mmol/L and <125 mmol/L respectively. Clinical information was gathered retrospectively.

**Results:**

486 patient episodes of hyponatraemia were included. The prevalence of hyponatraemia was 32.9%. The median age was 78 (min–max: 16–100) years and 239 (49.0%) were female. The median nadir sNa was 132 (min–max: 105–143) mmol/L. Eighty-seven (17.9%) and 48 (9.9%) patients had moderate and severe hyponatraemia. In cases of moderate and severe hyponatraemia, the most common cause of hyponatraemia was hypovolaemia (*n* = 33, 24.4%). Ninety-one patients (67.4%) with moderate and severe hyponatraemia had active treatment of hyponatraemia and 33 (24.4%) had input from a specialist service. The mean LOS was 15.0 (±22.5), 19.3 (±21.7) and 21.2 (±45.5) (*P* = 0.01) days in mild, moderate and severe hyponatraemia, respectively. Overall inpatient mortality was 7.0% (*n* = 34).

**Conclusions:**

Hyponatraemia is often incompletely investigated and suboptimally managed, with insufficient input from specialist services sought in a large tertiary hospital. Hyponatraemia therefore represents a potential intervention target to reduce inpatient morbidity, mortality and healthcare costs.

**Significance statement:**

Hyponatraemia is a common electrolyte disturbance among hospitalised patients, associated with increased morbidity, mortality and healthcare costs. In this retrospective study of medical inpatients at an Irish tertiary hospital, we found a high prevalence (32.9%) of hyponatraemia, with significant gaps in its investigation, diagnosis and management. Hypovolaemic hyponatraemia was the most frequent aetiology in moderate-to-severe cases, yet diagnostic tools and volume assessments were often underutilised. Suboptimal treatment approaches, including low rates of fluid restriction for SIADH, were evident. Our findings highlight the need for improved education, protocolised care and early specialist involvement to enhance outcomes. These results are generalisable to other centres, emphasising hyponatraemia as a key target for improving inpatient care and reducing healthcare costs.

## Introduction

Hyponatraemia, defined as a serum sodium (sNa) less than 135 mmol/L, is the most common electrolyte abnormality encountered in hospitalised medical patients (1, 2, 3). Hyponatraemia is consistently associated with increased inpatient morbidity and mortality, irrespective of the degree of severity (4, 5). Mortality rates in hyponatraemia have been demonstrated to be age-specific, with younger patients experiencing higher mortality compared with older patients with hyponatraemia (6, 7). In addition, mortality rates are influenced by the underlying aetiology (8). Cuesta *et al.* demonstrated higher mortality in hospitalised patients with hypervolaemic and hypovolaemic hyponatraemia when compared with those with syndrome of inappropriate antidiuretic hormone (SIADH), though mortality remained higher in those with SIADH than in normonatraemic controls (9).

Hyponatraemia is also associated with adverse healthcare metrics. A population-based cohort study of over 94,000 patients in Switzerland demonstrated higher 30-day readmission rates, prolonged length of stay (LOS), higher intensive care unit (ICU) admission and intubation rates, and increased risk of discharge to post-acute care facilities in hospitalised patients with hyponatraemia compared with normonatraemic patients (10). However, whether hyponatraemia itself directly contributes to these outcomes or simply reflects the severity of the underlying disease remains debated. Hoorn *et al.* suggested that hyponatraemia may act as a marker rather than a direct cause of morbidity and mortality, raising the question of whether improved patient outcomes are due to correcting the hyponatraemia itself or due to management focused primarily on the underlying pathology (11).

Signs and symptoms associated with hyponatraemia are variable. Acute severe hyponatraemia is a medical emergency with the potential to result in cerebral oedema, increased intracranial pressure, brain herniation and death (1, 12, 13, 14). Chronic hyponatraemia is also associated with adverse outcomes, including cognitive impairment, gait instability, falls, osteoporosis and increased fracture risk (14, 15, 16, 17, 18, 19). Treatment and resolution of chronic hyponatraemia have been shown to improve some of these outcomes, including gait, reaction times, cognitive scores and overall mortality (5, 14, 20, 21).

Despite the clinical importance of hyponatraemia, numerous studies have highlighted that hyponatraemia is often incompletely investigated (22, 23, 24). An observational study of the multinational hyponatraemia registry performed in 2015 demonstrated that in over 5,000 patients with hypervolaemic and euvolaemic hyponatraemia (defined as an sNa <130 mmol/L) fewer than 50% participants had appropriate laboratory testing for the diagnosis of SIADH, and correction rates were higher in those who had such investigations performed (24).

Consensus guidelines for the evaluation and management of hyponatraemia have been developed by interdisciplinary professional bodies in both the United States and Europe (1, 3). The aims of this audit were to evaluate the prevalence of hyponatraemia in adult medical patients (surgical, dialysis and oncology patients excluded) admitted through the emergency department in a large tertiary centre, compliance with the Clinical Practice Guidelines by Spasovski *et al.* and expert panel recommendations by Verbalis *et al.* regarding the evaluation and management of hyponatraemia, as well as LOS and mortality in this cohort (1, 3).

## Methods

This was a retrospective single-centre observational study conducted in a tertiary-level Irish hospital. Patients admitted under the adult (age ≥16 years) medical directorate with hyponatraemia (sNa <135 mmol/L) or who developed hyponatraemia during their inpatient stay over a 2-month period were included for analysis. Surgical, haematology, oncology, paediatric, obstetrics and end-stage renal disease patients were excluded. Patients were classified into groups based on the biochemical severity of the nadir sNa (mild, moderate or severe) as defined by the 2014 European Clinical Practice Guidelines (1). Mild, moderate and severe hyponatraemia were defined as sNa between 130 and 134 mmol/L, 125–129 mmol/L and less than 125 mmol/L respectively. We collected limited data on those with mild hyponatraemia and more detailed information on those with moderate and severe hyponatraemia, as morbidity and mortality have been demonstrated to be higher in these groups (4).

Patients admitted between February 1st and March 30th 2022 were identified through locally stored admission records. Laboratory results were collected from the Dedalus Clinical Manager (iCM) laboratory records software. Information on the aetiology of hyponatraemia, volume status classification (hypovolaemic, euvolaemic and hypervolaemic) and treatments initiated were collected from medical and prescription charts. Active treatments included intravenous 0.9 or 3% saline, fluid restriction, diuresis, discontinuation of causative medication, tolvaptan or demeclocycline. Clinical information was used to calculate the Charlson comorbidity index (CCI), a validated tool used to predict 10-year mortality in patients with multiple comorbidities. The CCI score is derived from the cumulative sum of the number and weighted severity of various comorbid conditions, with higher scores indicating an increased risk of mortality (25). Chronic hyponatraemia was defined as a previous episode of hyponatraemia (sNa <135 mmol/L) within the 2 years before admission. Hyponatraemia overcorrection was defined as a rise of sNa >10 mmol/L in 24 h. Details on osmotic demyelination were collected from electronic and paper medical records. Specialist referral was defined as a formal consultation to endocrinology or nephrology services.

Categorical variables are presented as numbers and percentages (%). Continuous variables are presented as mean ± standard deviation (SD) for normally distributed data or median and minimum–maximum (min–max) if otherwise. The independent *t*-test and one-way analysis of variance (ANOVA) were used to compare statistical differences of means between groups. The chi-squared test was used to compare categorical data. Pearson correlation and logistic regression analysis were used to explore associations between inpatient mortality, LOS and achievement of sNa >130 mmol/L on discharge and reported as correlation coefficient (r), odds ratios (ORs) and 95% confidence intervals (95% CIs) respectively. Variables of interest included nadir sNa, age, volume category, active treatment, ascertainment of urine biochemistry and specialist input. Statistical significance was set at *P* < 0.05. Statistical analyses were performed using Statistical Package for the Social Sciences (SPSS) version 27 (IBM Corporation, USA).

Data collection was undertaken between January and May 2023. This study was approved by the Clinical Research and Ethics Committee at University College Cork (Reference number: ECM4(s) 01/11/2022).

## Results

**Figure 1:**
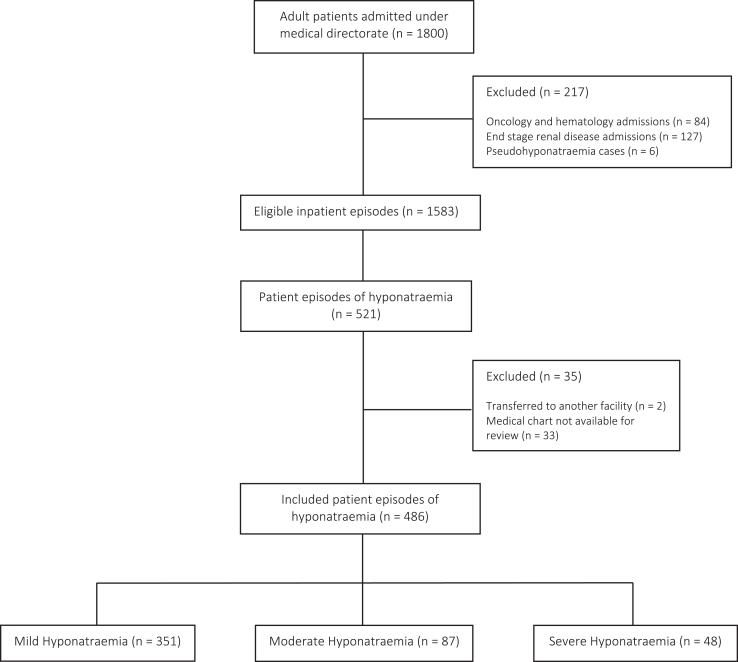
Flowchart of patient inclusion.

Of the 1,800 patients admitted under the medical directorate over the 2-month period, 521 patient episodes of hyponatraemia were identified in 512 patients, as shown in Fig. 1. The prevalence of hyponatraemia was 32.9%. Thirty-five (6.7%) cases were excluded from the final analysis as care was transferred to another facility or medical information was not available for review.

In total, 486 patient episodes of hyponatraemia were included: 351 (72.2%) with mild hyponatraemia, 87 (17.9%) with moderate hyponatraemia and 48 (9.9%) with severe hyponatraemia. The median nadir sNa was 132 mmol/L (min–max: 132). The median age was 78 (min–max: 16–100) years and 239 (49.0%) were female. Chronic hyponatraemia was documented in 352 patient episodes (72.1%). Demographic data are outlined in Table 1.

**Table 1:** Baseline characteristics.

	All (*n* = 486)	Mild (*n* = 351)	Moderate (*n* = 87)	Severe (*n* = 48)	*P* value
Age, years median (min–max)	78 (16–100)	78 (16–97)	79 (35–100)	75 (16–98)	0.09
Female, *n* (%)	239 (49.0)	166 (47.3)	46 (52.9)	27 (56.3)	0.43
sNa on admission, mmol/L median (min–max)	133 (107–143)	133 (130–143)	129 (125–137)	122 (107–142)	**<0.001**
Nadir sNa, mmol/L median (min–max)	132 (105–134)	133 (130–134)	128 (125–129)	120 (105–124)	**<0.001**
sNa on discharge or death, mmol/L median (min–max)	135 (120–160)	136 (120–169)	134 (125–142)	132 (120–140)	**<0.001**
sNa >130 on discharge, *n* (%)	454 (93.0)	349 (99.4)	72 (82.6)	33 (68.8)	**<0.001**
Chronic hyponatraemia, *n* (%)	352 (72.1)	255 (72.6)	65 (74.7)	32 (66.7)	0.88
LOS, days mean (SD)	15.0 (22.5)	13.1 (17.2)	19.3 (21.7)	21.2 (45.5)	**0.01**
Inpatient mortality, *n* (%)	34 (7.0)	27 (7.7)	4 (4.6)	3 (6.3)	0.56

*P* values represent the comparison of mild, moderate and severe groups; bold indicates statistical significance; LOS, length of stay; SD, standard deviation.

Further data on moderate and severe hyponatraemia are shown in Table 2. Hypovolaemic hyponatraemia was the most common cause of hyponatraemia in both moderate and severe hyponatraemia (*n* = 30, 22.2%), however a volume assessment was not documented in 50 (37.0%) cases. Twenty cases (14.8%) were diagnosed as SIADH hyponatraemia.

**Table 2:** Characteristics of moderate to severe hyponatraemia.

	Moderate hyponatraemia (*n* = 87)	Severe hyponatraemia (*n* = 48)	Moderate and severe hyponatraemia (*n* = 135)	*P* value
Volume category, *n* (%)				
Euvolaemic	15 (17.2)	11 (22.9)	26 (19.3)	0.09
Hypovolaemia	18 (20.7)	15 (31.3)	33 (24.4)	
Hypervolaemic	13 (14.9)	10 (20.8)	23 (17.0)	0.97^‡^
Not documented	41 (47.1)	12 (25.0)	53 (39.3)	
Paired biochemistry at time of nadir hyponatraemia, *n* (%)				
Urine sodium	23 (26.4)	33 (68.8)	56 (41.5)	**<0.001**
Urine osmolality	24 (27.5)	38 (79.2)	62 (45.9)	**<0.001**
Serum osmolality	23 (26.4)	35 (72.9)	58 (43.0)	**<0.001**
Serum glucose	50 (57.5)	35 (72.9)	85 (63.0)	0.08
08:00 h serum cortisol	17 (36.2)	24 (50.0)	41 (30.4)	**<0.001**
Thyroid function tests	56 (64.4)	37 (77.1)	93 (68.9)	0.13
Aetiology of hyponatraemia, *n* (%)				
Hypovolaemia	19 (21.8)	11 (22.9)	30 (22.2)	**0.02**
CCF	8 (9.2)	4 (8.3)	12 (8.8)	
Diuretic induced	4 (4.6)	6 (12.5)	10 (7.4)	0.38^‡^
SIADH	8 (9.2)	12 (25)	20 (14.8)	
Decompensated CLD	3 (3.4)	1 (2.1)	4 (3.0)	
Alcohol excess	2 (2.3)	1 (2.1)	3 (2.2)	
Adrenal insufficiency	2 (2.3)	2 (4.2)	4 (3.0)	
Primary polydipsia	0 (0)	2 (4.2)	2 (1.5)	
Not documented	41 (47.1)	9 (18.8)	50 (37.0)	
ICU admission, *n* (%)				0.94
For hyponatraemia	0 (0)	1 (2.1)	1 (0.7)	
For other indications	2 (2.3)	0 (0)	2 (1.5)	
Treatment administered, *n* (%)*				**<0.001**
No specific treatment	40 (46.0)	4 (8.3)	44 (32.6)	
0.9% saline infusion	34 (39.4)	20 (41.7)	54 (40.0)	
Hypertonic saline	0 (0)	1 (2.1)	1 (0.7)	
Fluid restriction	3 (3.4)	15 (31.3)	18 (13.3)	
Tolvaptan	0 (0)	0 (0)	0 (0)	
Demeclocycline	0 (0)	0 (0)	0 (0)	
Diuresis	8 (9.2)	3 (6.3)	11 (8.1)	
Discontinuation of potentially causative drug	6 (6.9)	8 (16.7)	14 (10.4)	
>1 treatment administered, *n* (%)	8 (9.2)	17 (35.4)	25 (18.5)	**<0.001**
Overcorrection^†^	3 (3.4)	3 (6.4)	6 (4.4)	0.45
Clinical osmotic demyelination	0 (0)	0 (0)	0 (0)	NA
Referral to specialist service	13 (14.9)	20 (41.7)	33 (24.4)	**<0.001**
CCI median (min–max)	5 (1–12)	4 (0–13)	5 (0–13)	**0.04**

CCF, congestive cardiac failure; CLD, chronic liver disease; SIADH, syndrome of inappropriate antidiuretic hormone; SSRI, selective serotonin reuptake inhibitor; TCA, tricyclic antidepressant; CNS, central nervous system; ICU, intensive care unit; CCI, Charlson comorbidity index.

*P* value represents a comparison of moderate hyponatraemia vs severe hyponatraemia. Bold indicates statistical significance.

* Patients could have more than one treatment administered.

^†^ Overcorrection defined as a rise of sNa >10 mmol/L in 24 h.

^‡^ *P* value when undocumented cases were excluded.

Urinary sodium concentration was obtained in 23 (26.4%) and 33 (68.8%) cases of moderate and severe hyponatraemia respectively. Urinary osmolality was obtained in 24 (27.5%) and 38 (79.2%) cases of moderate and severe hyponatraemia respectively. Serum osmolality was obtained in 58 (43.0%) cases of moderate and severe hyponatraemia. Of those labelled SIADH hyponatraemia, 11 (55.0%) and 15 (75.0%) had serum cortisol and thyroid function tests measured respectively.

Ninety-one patients (67.4%) with moderate-severe hyponatraemia had active treatment of hyponatraemia and 33 (24.4%) had input from a specialist service. The median time from nadir sNa to specialist referral was 1 day (min–max: 0–62). In cases of SIADH hyponatraemia, 0.9% saline was the most administered treatment (*n* = 9, 45.0%), followed by fluid restriction in six cases (30.0%). Hypertonic saline was administered in one case of severe hyponatraemia (nadir sNa 119 mmol/L). There were six cases (4.4%) of hyponatraemia overcorrection in those with moderate-severe hyponatraemia; however, there were no cases of clinical osmotic demyelination.

The overall mean LOS was 15.0 (±22.5) days, with 13.1 (±17.2), 19.3 (±21.7) and 21.2 (±45.5) days in mild, moderate and severe hyponatraemia respectively (*P* = 0.01). LOS and nadir sNa were negatively correlated (*r* = −0.14, *P* = 0.002). In those with moderate-severe hyponatraemia, LOS and nadir sNa remained negatively correlated when corrected for age and CCI (*r* = −0.19, *P* = 0.03). There was no statistically significant difference between LOS in those who received active treatment for hyponatraemia and those who did not (25.7 days (±50.9) days versus 14.9 days (±23.0), *P* = 0.18) or between those who received input from a specialist service and those who did not (26.1 (±53.2) days versus 20 (40.4) days, *P* = 0.56).

Ninety-three percent (*n* = 454) of the overall cohort had a sNa >130 mmol/L on discharge. Of these, 99.4, 82.6 and 68.6% had mild, moderate and severe hyponatraemia respectively. In those over the age of 65 years with moderate-severe hyponatraemia (*n* = 97), 74 (76.3%) patients had a sNa >130 mmol/L on discharge. In those with moderate-severe hyponatraemia, active treatment of hyponatraemia, referral to a specialist service or ascertainment of urine biochemistry did not increase attainment of sNa >130 mmol/L on discharge (OR 1.28, 95% CI 0.58–2.83; OR 1.83, 95% CI 0.68–4.87 and OR 0.62, 95% CI 0.27–1.42 respectively).

Overall inpatient mortality was 7.0%, with no statistical significance between groups (*P* = 0.56). In those with moderate-severe hyponatraemia, there was no significant association between age, nadir sNa, gender, volume category, CCI and mortality risk. A history of chronic hyponatraemia was associated with lower mortality (OR 0.07, 95% CI 0.01–0.69, *P* = 0.02). Active treatment and specialist input had no impact on mortality risk (OR 0.56, 95% CI 0.08–3.90 and OR 1.45, 95% CI 0.19–11.22, respectively).

## Discussion

In this study of hospitalised medical inpatients in an Irish tertiary centre, the overall prevalence of hyponatraemia, as defined by sNa <135 mmol/L, was 32.9% over a 2-month period. The prevalence of hyponatraemia in hospitalised patients in the literature is variable depending on the cohort included and sNa cut-offs applied (3). Our results, however, are consistent with previous reports using an sNa cut-off between 135 and 138 mmol/L, underscoring the importance of hyponatraemia being the most common electrolyte disorder in hospitalised patients (26, 27, 28).

Hypovolaemic hyponatraemia was the most common aetiology of hyponatraemia (22.2%) in moderate-severe cases, while 14.8% patients were diagnosed with SIADH hyponatraemia. Real-world data from a Swiss cohort study are consistent with our results, whereby 11.9% medical inpatients had a coded diagnosis of SIADH (10). In contrast, an Irish prospective study from Cuesta *et al.* reported a higher incidence of euvolaemic hyponatraemia (46.6%), with the majority of cases representing SIADH versus hypovolaemia (32.6%) and hypervolaemia (20.8%) (9). In this study, senior investigators performed volume assessments, likely accounting for the higher and potentially more accurate characterisation of euvolaemia and SIADH. In our study, 39.3% patients in the moderate to severe hyponatraemia group did not have volume status defined and documented, suggesting that, in practice, volume assessment and classification of hyponatraemia are often suboptimal.

Our findings highlight poor adherence to guideline-recommended diagnostic pathways for hyponatraemia, consistent with real-world data showing persistent gaps between best practice and routine care (23, 29). Consensus guidelines recommend assessment of serum and urine osmolality, urine sodium and volume status on initial evaluation, yet in our study, only 41.5 and 45.9% cases with moderate to severe hyponatraemia had urine sodium and osmolality measured, while 58% had serum osmolality assessed, and volume status was recorded in 60.7% cases (1, 3). These findings align with data from multiple centres reporting inadequate investigation, with studies reporting measurement of serum osmolality, urine sodium and urine osmolality in 26–39, 10–47 and 27–61% cases, respectively (23, 30, 31, 32, 33). Proper diagnostic workup is crucial, as obtaining appropriate laboratory investigations has been associated with higher success rates in correcting hyponatraemia, while misdiagnosis often leads to inappropriate treatment and can worsen patient outcomes (24, 29, 30). In our cohort, a diagnosis was coded in 63% cases, meaning that a significant proportion of patients were treated without understanding the underlying cause. Addressing these gaps requires clinical leadership from expert physicians to drive adherence to guidelines and change clinical attitudes (29). Studies show that expert input significantly improves diagnostic accuracy and patient outcomes, yet access remains limited (23, 31). In addition, artificial intelligence-driven decision-support systems could aid clinicians in making timely and evidence-based decisions (29). Implementing these interventions through education, structured multidisciplinary involvement and technology integration is essential to improve the diagnosis and management of hyponatraemia.

Fluid restriction is the first-line treatment for SIADH, as recommended by both European and US consensus guidelines, while urea and vasopressin receptor antagonists are reserved as second-line therapy (1, 3). In our cohort, 30% SIADH cases were managed with fluid restriction, with no patients receiving pharmacotherapy. Notably, 40% SIADH cases were treated with 0.9% saline administration. This finding is consistent with Thorpe *et al.* who reported that 0.9% saline was the most commonly used treatment for SIADH-related hyponatraemia (6).

These results suggest that suboptimal SIADH management is not unique to our centre but may reflect broader challenges, such as gaps in clinician education, a lack of standardised treatment protocols, or delays in obtaining diagnostic results necessary for timely diagnosis. The complete absence of pharmacotherapy in our cohort highlights the limited availability of tolvaptan, urea and demeclocycline experienced in many hospitals due to regulatory restrictions, cost barriers and clinician awareness. Addressing this issue requires a shift in approach to ensure that at least one pharmacological option is accessible in all healthcare settings. Promoting cost-effectiveness research and increasing clinician awareness can further support formulary inclusion and broader use, ensuring more consistent and effective management of SIADH.

Overall mortality in our cohort was 7.0%. We did not include a comparator group with normonatraemic patients to examine differences in inpatient mortality; however, the increased mortality risk associated with hyponatraemia compared with normonatraemic controls has been extensively reported in the literature (4, 5, 34, 35). In addition, the Irish national mortality rate for emergency hospitalised inpatients was reported as 2.8% in 2022 (36). In our study, there was no significant association between nadir sNa and mortality risk. These findings contrast with a meta-analysis of over 850,000 patients demonstrating an inverse correlation between sNa and mortality risk (5), while other studies have reported a paradoxical fall in mortality with declining sNa (4, 34). Given that age, CCI and gender distributions were comparable between groups, it is possible that additional confounders – such as comorbidity burden not fully captured by the CCI or differences in treatment interventions – may have influenced outcomes in our cohort. Furthermore, our exclusion of surgical admissions, haematological, oncological and end-stage renal disease patients may have impacted our findings, as these populations are variably included in other case–control studies examining hyponatraemia and mortality (4, 34, 37, 38, 39).

Interestingly, in our study, a history of chronic hyponatraemia, defined as a previous episode of hyponatraemia (sNa <135 mmol/L) in the previous 2 years, was associated with decreased mortality risk (OR 0.07, 95% CI 0.006–0.69, *P* = 0.02). Cuesta *et al.* reported that, in a study of 1323 admissions with hyponatraemia, overall mortality was lower in cases of SIADH hyponatraemia when compared with hypervolaemic and hypovolaemic cases (9). The authors hypothesised that mortality in hypervolaemic and hypovolaemic hyponatraemia was secondary to the underlying disease process. Potentially, a large number of our cohort had undiagnosed SIADH hyponatraemia manifesting as recurrent episodes of hyponatraemia, contributing to a lower mortality observed in those with chronic hyponatraemia.

The overall mean LOS was 15.0 days (±22.5), with LOS found to be negatively correlated with nadir sNa. This contrasts with the Irish national average LOS for emergency inpatients in 2022, which was reported as 6.7 days (36). In those with moderate-severe hyponatraemia, LOS and nadir sNa remained negatively correlated when corrected for age and CCI, suggesting that prolonged LOS in hyponatraemia cannot be attributed to age and comorbidity burden. This is consistent with other studies. A large retrospective cohort study in the United States of over 10,000 episodes of hyponatraemia on admission demonstrated that hyponatraemia was independently associated with increased need for ICU admission and mechanical ventilation, higher mortality, increased hospital costs and LOS (40). Amin *et al.* in addition to reporting increased hospital LOS, cost and ICU admission rates with hyponatraemia, reported that hyponatraemia was associated with a 15.0% increase in 30-day readmission rates (41). Therefore, hyponatraemia is associated with increased healthcare costs.

In our cohort, 25% patients over the age of 65 years with moderate-severe hyponatraemia were discharged with persistent hyponatraemia (sNa ≤130 mmol/L). This is significant in light of the complications associated with chronic hyponatraemia, including cognitive impairment, gait instability, falls and fractures, which may further add to the healthcare cost burden (14, 15, 16, 17, 18). While observational data have indicated that resolution of hyponatraemia generates improvement in functional and neurocognitive domains, further prospective randomised trials are required to explore these and other clinically relevant outcomes (14, 20, 42).

We were surprised to note that referral to a specialist service (endocrinology or nephrology) did not improve inpatient mortality, LOS or attainment of sNa >130 mmol/L on discharge. This may be due to the low number of cases for whom specialist input had been sought (33 cases, 24.4%). In addition, it is our experience that specialist input is only sought in difficult or refractory cases whereby first-line treatments and investigations failed to improve sNa. In contrast, a prospective controlled intervention study in the United Kingdom demonstrated that prompt endocrine intervention in cases of SIADH hyponatraemia resulted in a more rapid rise in sNa, higher rates of sNa >130 mmol/L on discharge, and decreased mean length of hospital stay (10.9 vs 14.5 days; *P* < 0.01) (43). Similarly, a large Irish observational study performed over 10 years demonstrated that increased hospital education, structured specialist input and active management of severe hyponatraemia were associated with a reduction in mortality, higher sNa on discharge and a reduction in LOS (21).

This study has some limitations. It was performed in a single centre, which facilitated accurate data collection; however, our site is also a large neurosurgical, renal and oncology centre. We excluded these cases in order to reflect the disease spectrum in general medical patients. In addition, the retrospective observational nature of the study has limitations, as well as a lack of a control group. Much of our data were analysed according to documentation by the treating team; therefore, treatment strategies and diagnoses may have been discussed and not recorded.

In conclusion, hyponatraemia is frequently encountered in clinical practice and is associated with considerable morbidity, mortality and healthcare costs (1, 2, 4, 5, 10). Our findings highlight gaps in investigation, suboptimal management and limited specialist input, underscoring the need for targeted interventions. Emerging therapies, including tolvaptan, urea and sodium-glucose cotransporter-2 inhibitors, offer new treatment options, particularly in cases where fluid restriction is impractical or ineffective (8, 19, 44). Updated guidelines are needed to incorporate these advancements and provide a standardised, evidence-based approach to diagnosis and treatment. Future recommendations should emphasise early and thorough assessment, appropriate therapeutic selection, and the integration of specialist input to optimise patient outcomes (8). Large, multicentre prospective controlled studies should further refine these guidelines to optimise real-world outcomes and cost-effectiveness.

## Declaration of interest

The authors declare that there is no conflict of interest that could be perceived as prejudicing the impartiality of the work reported.

## Funding

This work did not receive any specific grant from any funding agency in the public, commercial or not-for-profit sector.

## Author contribution statement

All authors contributed to the study conception and design. Material preparation and data collection were performed by Aoife Courtney, Lok Yi Joyce Tan, Ali Elsheikh, Faye O’Donovan, Iman Faez, Rachel Riegel and Tara O’Sullivan. Data analysis was performed by Aoife Courtney and Oratile Kgosidialwa. The first draft of the manuscript was written by Aoife Courtney and all authors commented on previous versions of the manuscript. All authors read and approved the final manuscript.

## Data availability

Data supporting this study are stored locally and can be made available on request.

## Ethics approval

This study was approved by the Clinical Research Ethics Committee at University College Cork.
